# BioGlue® manifesting as a subaortic floating object seen during ventricular septal rupture surgery: a case report

**DOI:** 10.1186/s40981-020-00332-9

**Published:** 2020-04-14

**Authors:** Kenzaburo Sugimoto, Mamoru Kadosaki, Sho Nakata, Kei Aizawa, Koji Kawahito, Mamoru Takeuchi

**Affiliations:** 1 Department of Anaesthesiology and Critical Care Medicine, Jichi Medical University, 3311-1, Yakushiji, Shimotsuke City, Tochigi 329-0498 Japan; 2 Department of Surgery, Division of Cardiovascular Surgery, Jichi Medical University, 3311-1, Yakushiji, Shimotsuke City, Tochigi 329-0498 Japan

**Keywords:** BioGlue, Biological glue, Ventricular septal rupture, Embolism

To the Editor,

BioGlue (Cryolife Inc., Kennesaw, GA, USA) is a biological adhesive that supports hemostasis of surgical suture lines. It is mainly used during aortic dissection repair [1]. For the past two decades, the glue has come into more common during intracardiac surgery, including ventricular septal rupture (VSR) surgery [1]. Although the glue’s contact with the bloodstream likely enhances the risk of embolic events [2], there have been no reports of BioGlue causing an embolic event during VSR surgery. We report a case of VSR surgery during which we detected a subaortic floating object, later identified as a BioGlue clot.

A 72-year-old man presented a 1-week history of worsening tiredness. On admission, he was found to have myocardial infarction. Percutaneous intervention was undertaken, and an intra-aortic balloon pump (IABP) was placed. Approximately 5 h later, severe hypotension occurred, and echocardiography revealed VSR. He underwent urgent septal patch closure. The incision was made in the right ventricular wall. BioGlue was sprayed into the space between the double patches, from the right ventricle toward the left ventricle. Before he was weaned from cardiopulmonary bypass, transesophageal echocardiography (TEE) showed a subaortic floating object that had not been seen preoperatively (Fig. 1). TEE also revealed exacerbated mitral regurgitation, which prompted the surgeon to replace the mitral valve. During this additional operation, the subaortic object was removed and diagnosed as a BioGlue based on its appearance (Fig. 2). The patient was weaned from cardiopulmonary bypass after confirming a lack of abnormal objects by TEE, and the surgery completed uneventfully. The patient was extubated on postoperative day 8 and weaned from the IABP the following day. Thereafter, however, his postoperative course became complicated owing to right heart failure, and he died on postoperative day 29.

**Fig.1:**
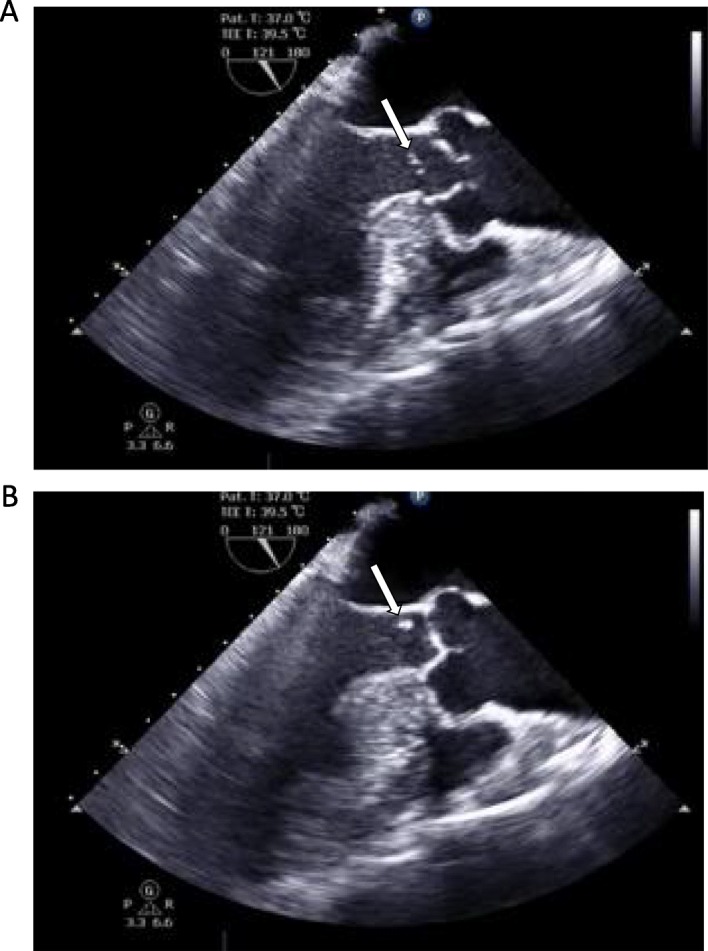
A subaortic floating object detected by transesophageal echocardiography. A subaortic membranous object (white arrow) moves up and down with a cardiac cycle. 1**a**, systolic phase; 1**b**, diastolic phase

**Fig. 2:**
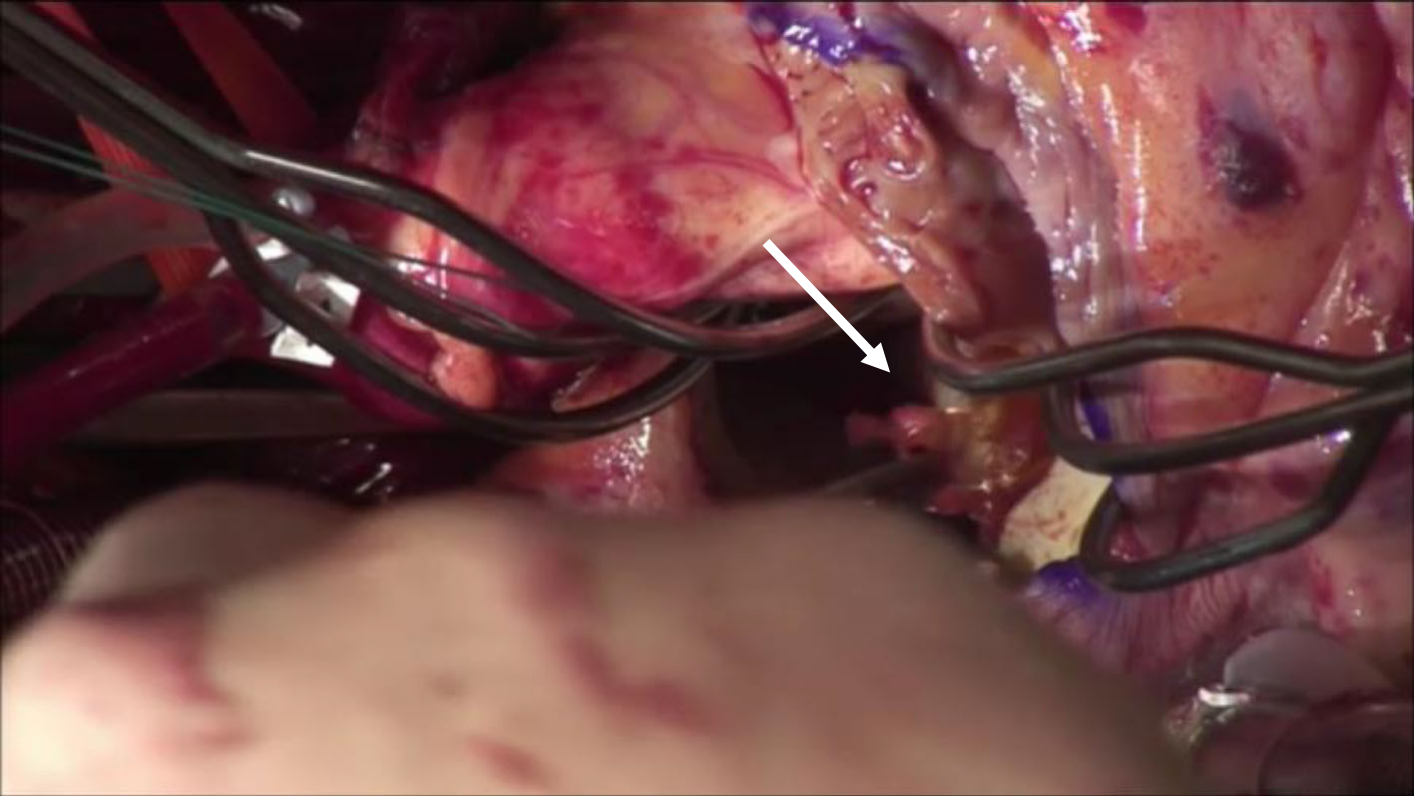
The BioGlue in the surgical field. The biological glue (white arrow) under the aortic valve was removed

It has been previously reported that the BioGlue was responsible for lower extremity or coronary artery embolism when it had been applied to the suture line between the aorta and the graft outside the heart [3, 4]. Cryolife Inc. warns that the Bioglue should not be applied within the intracardiac cavity because the glue is more likely to cause an embolism when applied to an intracardiac cavity than when used at extracardiac sites [2]. However, shunt recurrence between the patch and the septal myocardium is one of several lethal complications after VSR surgery, and surgeons occasionally use the glue to reinforce the suture line between septum and the patch [1, 5, 6]. Double patch repair is frequently adopted, and in those cases BioGlue is often sprayed into the space between the two patches so as not to allow the glue to contact the systemic circulation [5, 6]. If the anastomotic site has loosened, glue leakage and contact with the bloodstream can occur. It is therefore recommended that anesthesiologists confirm the surgeons about the use of BioGlue. In cases where it is used, a careful TEE examination, particularly around the patch, is required to find any abnormal objects, thereby helping to avoid postoperative systemic embolism.


**Additional file 1: Video S1.**




**Additional file 2: Video S2**.


## Data Availability

The datasets described in this article are included within article.

## Abbreviations

VSR: Ventricular septal rupture
TEE: Transesophageal echocardiography
IABP: Intra-aortic balloon pump

## References

[1] Fink D, Klein JJ, Kang H (2004). Application of biological glue in repair of intracardiac structural defects. Ann Thorac Surg..

[2] CryoLife Inc. BioGlue Surgical Adhesive; Medical Information; IFU for BioGlue Surgical Adhesive (USA). https://www.cryolife.com/wp-content/uploads/2019/08/L6312.010_IFU_BioGlue_Syringe.pdf. Accessed 2 Feb 2020.

[3] Mahmood Z, Cook DS, Luckraz H (2004). Fatal right ventricular infarction caused by Bioglue coronary embolism. J Thorac Cardiovasc Surg..

[4] El Feghaly M, Chahine E, Abi Ghanem M (2011). Acute limb ischemia due to embolisation of biological glue 45 days after surgery. Eur J Vasc Endovasc Surg..

[5] Asai T (2016). Postinfarction ventricular septal rupture: can we improve clinical outcome of surgical repair?. Gen Thorac Cardiovasc Surg..

[6] Higashi R, Matsumura Y, Yamaki F (2013). Posterior ventricular septal perforation: sandwich technique via right ventriculotomy using BioGlue. Gen Thorac Cardiovasc Surg..

